# Systematic review of probiotics for the treatment of community-acquired acute diarrhea in children

**DOI:** 10.1186/1471-2458-13-S3-S16

**Published:** 2013-09-17

**Authors:** Jennifer A Applegate, Christa L Fischer Walker, Ramya Ambikapathi, Robert E Black

**Affiliations:** 1 Department of International Health, The Johns Hopkins Bloomberg School of Public Health, Baltimore, MD 21205, USA

## Abstract

**Background:**

Oral rehydration salts (ORS), zinc, and continued feeding are the recommended treatments for community-acquired acute diarrhea among young children. However, probiotics are becoming increasingly popular treatments for diarrhea in some countries. We sought to estimate the effect of probiotics on diarrhea morbidity and mortality in children < 5 years of age.

**Methods:**

We conducted a systematic review of randomized controlled trials to estimate the effect of probiotic microorganisms for the treatment of community-acquired acute diarrhea in children. Data were abstracted into a standardized table and study quality was assessed using the Child Health Epidemiology Reference Group (CHERG) adaption of the GRADE technique. We measured the relative effect of probiotic treatment in addition to recommended rehydration on hospitalizations, duration and severity. We then calculated the average percent difference for all continuous outcomes and performed a meta-analysis for discrete outcomes.

**Results:**

We identified 8 studies for inclusion in the final database. No studies reported diarrhea mortality and overall the evidence was low to moderate quality. Probiotics reduced diarrhea duration by 14.0% (95% CI: 3.8-24.2%) and stool frequency on the second day of treatment by 13.1% (95% CI: 0.8 – 25.3%). There was no effect on the risk of diarrhea hospitalizations.

**Conclusion:**

Probiotics may be efficacious in reducing diarrhea duration and stool frequency during a diarrhea episode. However, only few studies have been conducted in low-income countries and none used zinc (the current recommendation) thus additional research is needed to understand the effect of probiotics as adjunct therapy for diarrhea among children in developing countries.

## Background

Diarrhea remains the second leading cause of death among children 1-59 months of age [1]. Currently, WHO recommends treatment with oral rehydration salts (ORS) and continued feeding for the prevention and treatment of dehydration, as well as zinc to shorten the duration and severity of the episode [2]. Probiotics are not recommended by WHO for the treatment of community-acquired acute diarrhea, though they are becoming increasingly popular in some countries [3].

Probiotics are non-pathogenic live microorganisms. When ingested, probiotics can survive passage through the stomach and small bowel [4]. They compete with enteric pathogens for available nutrients and bacterial adhesion sites, increase the acidity of the intestinal environment, synthesize compounds that destroy or inhibit pathogens, and may stimulate the host’s immune response to invading pathogens [4,5].

In previous meta-analyses of the efficacy of probiotic treatment for acute diarrhea in children, authors restricted their searches to specific probiotic strains [6-8]. A 2010 Cochrane systematic review of the use of probiotics for the treatment of acute diarrhea found a significant reduction in the mean duration of diarrhea (24.76 hrs; 95% CI 15.91 - 33.61 hrs) and stool frequency on the second day of treatment (mean difference 0.80; 95% CI 0.45 -1.14) [3]. In the Cochrane review, authors did not limit their searches to a particular strain, but included both adults and children in the study population and studies that limited inclusion to one etiology (e.g., only children with stools positive for rotavirus).

We sought to conduct a systematic review and meta-analysis of all probiotics for the treatment of community-acquired acute diarrhea specifically among children < 5 years of age. This systematic review was conducted to examine the efficacy of probiotics in diarrhea treatment and was designed to meet the needs of the Lives Saved Tool (*LiST*) [9].

## Methods

We conducted a systematic literature review to identify randomized controlled trials (RCT) of probiotics for the treatment of community-acquired acute diarrhea among children < 5 years of age. We employed the Child Health Epidemiology Reference Group (CHERG) guidelines [9] and searched all published literature from PubMed, Cochrane Library, WHO Regional Databases, Web of Science, Biosis, Popline, Global Health, Scopus, and Embase for relevant literature in all available languages published before December 1, 2012. We used various combinations of the Medical Subject Heading Terms (MeSH) and all fields search terms for *probiotics* and *diarrhea.* Given the wide variety of possible therapeutic probiotic microorganisms, we also searched using nomenclature variations of probiotic microorganisms (e.g., *Lactobacillus acidophilus*, *S. boulardii*, *etc.*)*.* If reports were unavailable for full-text abstraction, we made every effort to obtain the unpublished data from the authors. The complete search strategy is available in a WebAppendix (Additional file 1).

## Inclusion/Exclusion criteria

We included RCTs conducted among children < 5 years of age with acute diarrhea defined as ≥ 3 loose or watery stools per day, and a suitable control group. A suitable control group was defined as a group that was identical to the treatment group, but received a placebo and/or the appropriate standard of care for acute diarrhea in lieu of the probiotic. We sought a representative population of community-acquired diarrhea and thus excluded studies that: a) excluded all breastfed children; b) excluded specific types of diarrhea by etiology or only focused on a specific etiology; c) included children with a history of or current antibiotic use; or d) studies that did not evaluate probiotics alone. We included studies with at least 1 of the following outcomes: mortality, hospitalizations, severity (stool frequency on day 2, as a secondary measure of severity), or diarrhea duration.

## Abstraction and analysis

We abstracted all studies that met our inclusion/exclusion criteria into a standardized abstraction form (Additional file 2). We then organized abstracted data by outcome and probiotic microorganism. Abstracted variables included study design, probiotic definition and dosage, point estimates for both study arms, and relative outcome effect. Individual study arm characteristics are described in Table 1, including the probiotic agent, dosage (in colony-forming units (CFU) or milligrams) and duration of treatment (See Additional file 3 for the full version of Table 1). Based on the study characteristics, we evaluated the quality of evidence using the CHERG adaptation of the GRADE technique [9] (Tables 2 &3).

**Table 1:** Study characteristics

			Study arms			
	
		Intervention			Control	
	
Author	n	*Probiotic microorganism* (dosage)	Duration & method of treatment	n		Placebo
Boudraa [12]	56	*L. bulgaricus* &*S. thermophilus* (2x10^8^ CFU/g)	180 mL/kg/day infant formula given after initial oral rehydration	56		Infant formula acidified with lactic acid to match yogurt pH

Canani [13]	100	*Lactobacillus GG* (6x10^9^ CFU/dose)	2/day for 5 days in 20 ml water	92		Not described
	91	*S. boulardii* (5x10^9^ live organisms/dose)	2/day for 5 days in 20 ml water	92		
	100	*Bacillus clausii* (10^9^ CFU/dose)	2/day for 5 days in 20 ml water	92		
	97	*L. bulgaricus*, *L. acidophilus*, *S. thermophilus*, *B. bifidum* (5x10^8^- 10^9^ CFU/dose)	2/day for 5 days in 20 ml water	92		
	91	*Enterococcus faecium* (7.5x10^7^ CFU/dose)	2/day for 5 days in 20 ml water	92		

Cetina-Sauri [18]	65	*S. boulardii* (200 mg/dose)	Every 8 hours in 5ml of cold liquid	65		200mg glucose in 5ml cold liquid

Costa-Ribeiro [14]	61	*Lactobacillus GG* (10x10^9^ CFU/day)	1/day w/oral electrolyte solution	63		Inulin

Lee [15]	50	*Lyophilized L. acidophilus and B. infantis* (3x10^9^ CFU of each/day)	1/day for 4 days	50		Not described

Misra [16]	105	*Lactobacillus GG* (1x10^9^ CFU/dose)	1/day for 10 days	105		Identical placebo (crystalline microcellulose)

Rafeey [19]	40	*L. acidophilus* (5x10^9^ CFU/capsule)	2 capsules/ day	40		Not described

Veereman-Wauters [17]	Diarrhea duration: 25; Stool frequency: 22	*Lactobacillus GG*, *L. acidophilus*, *L. casei*, *L. plantarum*, *B. infantis* (3x10^9^ CFU/capsule)	3 capsules/ day for 9 days	22		Identical placebo (maltodextrine)

**Table 2:** Quality of evidence for treatment of diarrhea with probiotics for diarrhea duration and stool frequency on day 2

	Quality assessment					Summary of findings
	
				Directness		
		
No. of studies (study arms)	Design	Limitations	Consistency	Generalizability to population of interest	Generalizability to intervention of interest	Average percent difference (95% CI)
**Diarrhea duration**						
*Various probiotics:* low [12-17]						
6(10)	RCT	Different doses/day; variable treatment duration; not double blinded, small n, placebo not described (*-0.5*)	8/10 arms in the positive direction; 4/10 results statistically significant (*-0.5*)	Algeria, Italy, Brazil, Belgium, India, Taiwan (*-0.5*)	Mixtures prevent analysis of individual effect sizes, not enough data to make a statement about each probiotic strain (*-0.5*)	-14.0 (-24.2 – -3.8%)
*Lactobacillus rhamnosus GG:* moderate [13,14,16]						
3(3)	RCT	Different doses/day; variable treatment duration; not double blinded (*-0.5*)	3/3 studies in the positive direction; 1/3 study results statistically significant	Italy, Brazil, India	Generalizable	-16.0 (-53.9 – 22.0%)

**Stool frequency (Day 2)**						
*Various probiotics:* moderate/low [13,15,17-19]						
5(9)	RCT	Not double blinded, small n, placebo not described (*-0.5*)	5/9 arms in the positive direction; 4/9 results statistically significant (*-0.5*)	Italy, Iran, Mexico, Taiwan, Belgium	Mixtures prevent analysis of individual effect sizes, not enough data to make a statement about each probiotic strain (*-0.5*)	-13.1 (-25.3 – -0.8%)

**Diarrhea duration (study arms): ***LGG* (3), *S. boulardii* (1), *Bacillus clausii* (1), *Enterococcus faecium* (1), Mix A*(1), Mix B**(1), Mix C***(1), Mix D****(1)						
**Stool frequency (study arms): ***LGG* (1), *S. boulardii* (2), *Bacillus clausii* (1), *Enterococcus faecium* (1), *L. acidophilus* (1), Mix A*(1), Mix B**(1), Mix C***(1)						

*L. bulgaricus, *L. acidophilus*, *Streptococcus thermophilus*, *B. bifidum*

** L. acidophilus & Bifidobacteria infantis

*** LGG, *L. acidophilus*, *L. casei*, *L. plantarum*, *Bifidobacterium infantis*

****L. *bulgaricus & S. thermophilus*

**Table 3:** Quality of evidence for treatment of diarrhea with probiotics for hospitalizations

	Quality assessment					Summary of findings		
	
				Directness		No. of events		Effect
	
No. of studies (study arms)	Design	Limitations	Consistency	Generalizability to population of interest	Generalizability to intervention of interest	Intervention	Control	Relative Risk (95% CI)
**Diarrhea hospitailizations**								
*Various Probiotics:* Moderate/Low [13,17]								
2(6)	RCT	Not double blinded, small n, 5/6 study arms compared to same control; 0 results statistically significant (*-0.5*)	Homogeneous based on meta-analysis (p=0.735; I^2^=0%)	Italy & Belgium *(-0.5*)	Mixtures prevent analysis of individual effect sizes, not enough data to make a statement about each probiotic strain (*-0.5*)	16	20	0.81 (0.42 – 1.57)

**Hospitalizations (study arm): ***LGG* (1), *S. boulardii* (1), *Bacillus clausii* (1), *Enterococcus faecium* (1), Mix A* (1), Mix C*** (1)								

* *L. bulgaricus*, *L. acidophilus*, *Streptococcus thermophilus*, *B. bifidum*

*** LGG, *L. acidophilus*, *L. casei*, *L. plantarum*, *Bifidobacterium infantis*

For the analysis we grouped all studies by outcome. Given that the treatment effects may vary by probiotic organism, we grouped study results by probiotic strain used as the treatment agent where at least 3 studies were identified. We then performed subgroup analyses for the relevant outcomes.

To measure the relative effect we calculated the percent difference (I-C/C*100) for continuous outcomes. We calculated the percent difference with the estimated means and weighted each by the combined sample size of the intervention and control groups by study arm. In the case of multiple treatment groups and a single control group, we weighted each study arm by the intervention sample size and a proportion of the control group sample size. We then used the percent difference to calculate a weighted average. For studies that only presented median (IQR), we estimated the mean using a standard formula for studies with samples sizes greater than 25 [10]. We used a random effects meta-analysis to analyze discrete outcomes and reported the DerSimonian-Laird pooled relative risk and corresponding 95% confidence interval. The STATA 11 statistical software was used for all analyses [11].

## Results

**Figure 1:**
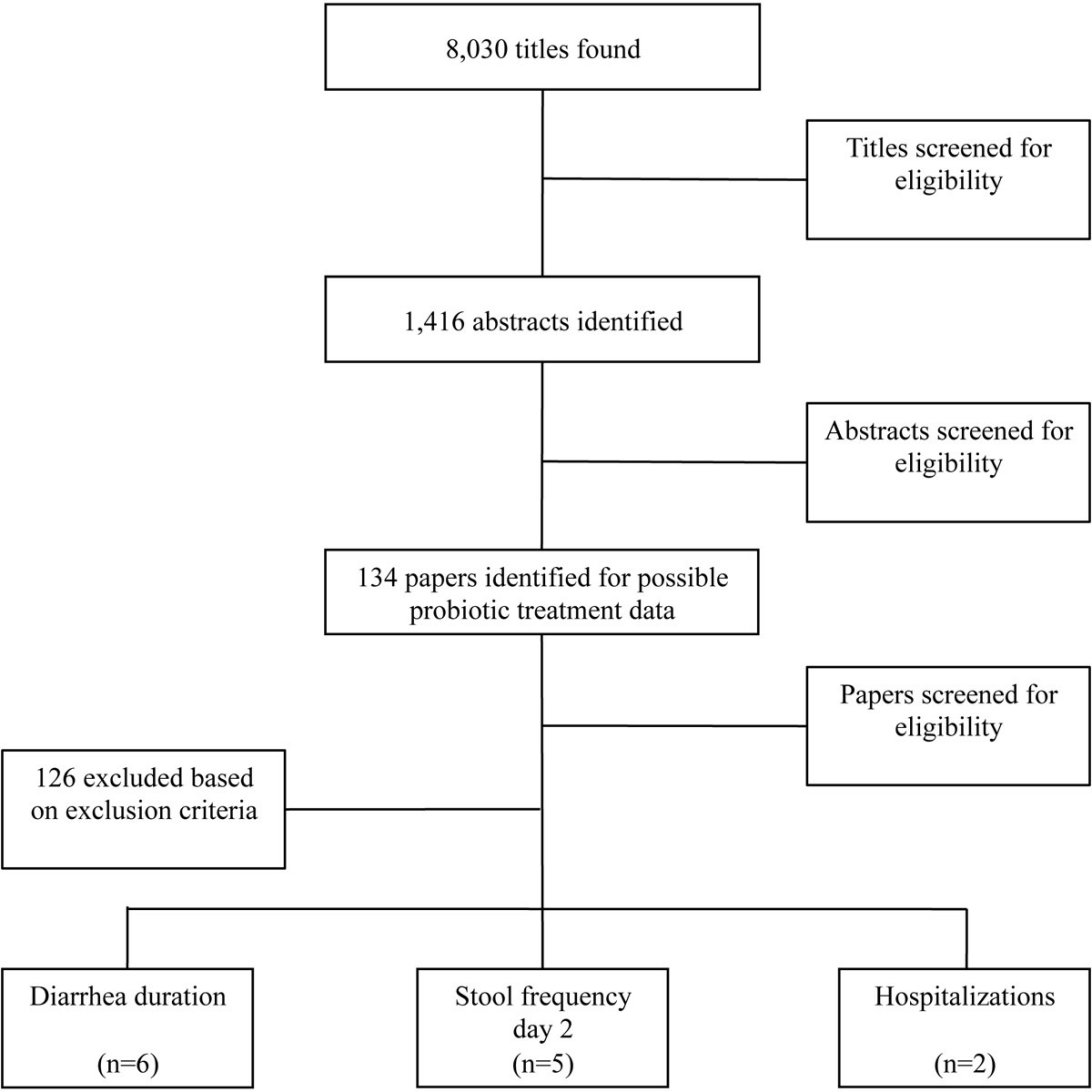
Results of literature search for studies on treatment of diarrhea with probiotics

We identified 8,030 titles from the literature search. After exclusion based on title and abstract, we obtained and reviewed 134 full papers and included 8 in the final database (Figure 1). Of these included papers, 6 studies included an outcome for diarrhea duration [12-17], 5 included stool frequency on day 2 [13,15,17-19] and 2 included a count of diarrhea related hospitalizations [13,17]. No studies included diarrhea mortality. All included studies were at least single-blinded RCTs; the researchers were blinded but in some cases caregivers were not [13,17]. Based on the combined study limitations and inconsistency of results, we determined the included studies to be of low to moderate quality according to GRADE guidelines [9] (Tables 2 &3).

**Figure 2:**
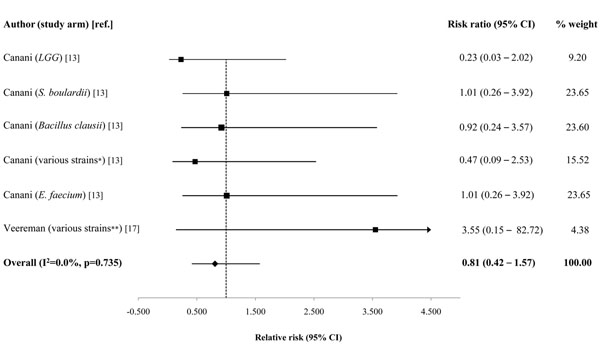
**Forest plot for the effect of probiotics as compared to control on diarrhea hospitalizations Legend:** * *L. bulgaricus*, *L. acidophilus*, *Streptococcus thermophilus*, *B. bifidum* ** *LGG*, *L. acidophilus*, *L. casei*, *L. plantarum*, *B. infantis*

Effect sizes varied widely across individual studies (Table 4). Based on the average percent difference, probiotics reduced diarrhea duration by 14.0% (95% CI: 3.8-24.2%) and stool frequency on day 2 of treatment by 13.1% (95% CI: 0.8 – 25.3%) (Table 2). We found no effect on diarrhea duration among the *Lactobacillus rhamnosus GG* (*LGG*) only group (16.0%; 95% CI -53.9 – 22.0%) (Table 2). There was no difference in the relative risk of hospitalization among children who received probiotics compared with placebo (RR=0.81; 95% CI: 0.42–1.57) (Table 3 & Figure 2).

**Table 4:** Percent difference and weight contributed by study and continuous outcome

Probiotic microorganism	Study arm by author	Percent difference	% Weight
**Diarrhea duration**			

*Lactobacillus rhamnosus GG*	Canani [13]	-32.0*	10.2
*Lactobacillus rhamnosus GG*	Costa-Ribeiro [14]	-2.1	10.6
*Lactobacillus rhamnosus GG*	Misra [16]	-9.5	18.0
*L. bulgaricus & S. thermophilus*	Boudraa [12]	-28.5^§^	9.6
*Saccharomyces boulardii*	Canani [13]	-9.1	9.4
*Bacillus clausii*	Canani [13]	2.2	10.2
*Enterococcus faecium*	Canani [13]	0.0	9.4
*L. bulgaricus*, *L. acidophilus*, *Streptococcus thermophilus*, *B. bifidum*	Canani [13]	-39.4*	9.9
*L. acidophilus & Bifidobacteria infantis*	Lee [15]	-13.9*	8.6
*Lactobacillus GG*, *L. acidophilus*, *L. casei*, *L. plantarum*, *Bifidobacterium infantis*	Veereman-Wauters [17]	-7.5	4.0

**Stool frequency (day 2)**			

*Lactobacillus rhamnosus GG*	Canani [13]	-20.0*	12.8
*Lactobacillus acidophilus*	Rafeey [19]	0.0	8.6
*Saccharomyces boulardii*	Canani [13]	0.0	11.9
*Saccharomyces boulardii*	Cetina-Sauri [18]	-14.2^§^	14.0
*Bacillus clausii*	Canani [13]	0.0	12.8
*Enterococcus faecium*	Canani [13]	0.0	11.9
*L. bulgaricus*, *L. acidophilus*, *Streptococcus thermophilus*, *B. bifidum*	Canani [13]	-20.0*	12.5
*L. acidophilus & Bifidobacteria infantis*	Lee [15]	-48.6*	10.8
*Lactobacillus GG*, *L. acidophilus*, *L. casei*, *L. plantarum*, *Bifidobacterium infantis*	Veereman-Wauters [17]	-16.7	4.7

Statistically significant differences between groups at the p<0.01 and p<0.05 levels indicated by (*) and (§) respectively for diarrhea duration and stool frequency

## Discussion

We conducted a systematic review of RCTs to estimate the effect of probiotic microorganisms for the treatment of community-acquired acute diarrhea in children. Results of this systematic review indicate that probiotics reduced stool frequency on the second day of treatment by 13.1%. When we combined all the study arms we found a 14.0% reduction in diarrhea duration among those who received probiotics compared to those who received placebo. Of the 10 study arms included in the analysis, only 1 *LGG* arm [13] and 3 probiotic mixtures [12,13,15] found a significant reduction in diarrhea duration with effect sizes of 32%, 28.5%, 39.4% and 13.9% respectively (Table 4).

Probiotics did not have an effect on the relative risk of hospitalization between children in the treatment and control groups. None of the included studies reported diarrhea deaths, thus we were limited to outcomes that reflected diarrhea morbidity. Based on the available data, relative risk of hospitalization was the best measure of severe morbidity, but this outcome had a limited number of events across the two included studies [13,17] (Table 3). None of the individual study arms reported a significant difference in hospital admissions between treatment and control groups, but studies were not powered for this outcome measure.

Despite a number of systematic reviews on the efficacy of probiotic treatment in infectious diarrhea, this is the first to apply the CHERG guidelines to estimate the effect of probiotic treatment on community-acquired acute diarrhea among children for inclusion in the *LiST* software. This review follows the CHERG systematic review methods required of all *LiST* interventions to estimate the effect of the intervention on cause-specific mortality [9]. The *LiST* tool is designed to provide international agencies and policymakers with evidence-based estimations of lives that could be saved with the scale-up of key interventions [20]. Thus, the implications of this review are important for programmatic and policy decisions in the management of childhood diarrheal disease in low- and middle-income countries (LMIC).

Our review indicates a reduction in diarrhea duration and stool frequency, which may be indicative of attenuated intensity of the intestinal infection [21]. These results are consistent with findings in previous systematic reviews [3,8]. Previous studies have shown that benefits of probiotics on diarrhea may be strain and/or etiology specific [6,13,22], meaning only certain strains may be efficacious in the treatment of a particular etiology of diarrhea. Prior systematic reviews have found larger effect sizes for probiotic treatment on diarrhea duration than what we report here [3,6], but they included studies among specialized populations such as young children with confirmed rotavirus diarrhea or populations of infants who were completely weaned. Given that the etiologies of most childhood diarrhea episodes in developing countries are not confirmed, we designed this review to include only studies that did not exclude based on etiology. To be programmatically relevant at the community/household level the intervention must demonstrate efficacy for episodes of non-specific origin.

As a secondary aim of this review, we evaluated the efficacy of individual probiotic strains for acute diarrhea treatment. We evaluated strain-specific differences in therapeutic properties for probiotics with 3 or more study arms. Based on the limited number of included studies and wide variation in probiotic organisms, we were only able to examine the specific pooled effect of *LGG* on diarrhea duration. These results were not found to be significant. Also, we were not able to separate the various mixtures of probiotics to evaluate the efficacy by individual strain or dose.

There are limitations associated with the studies we included and analysis as it pertains to the CHERG guidelines. Ideally, we would have included only community-based studies conducted in LMIC. Because we only found 5 studies from LMIC and of these 3 reported diarrhea duration [12,14,16], 2 reported stool frequency on day 2 [18,19], and none reported diarrhea hospitalizations, we chose to include studies from high-income countries [13,15,17]. No studies were conducted in the community, all were in outpatient or hospital settings, and thus results may not be generalizable to home-based treatment of diarrhea episodes. All included RCTs had a control group that received a blinded placebo and/or standard of care, but the placebo substances were not standardized across studies. We controlled for potential confounding effects of varying placebos by excluding studies that used placebos with potential therapeutic properties in treating diarrhea (e.g., prebiotics or calcium supplements).

In addition, WHO has recommended low osmolarity ORS, continued feeding, and zinc for the treatment of diarrhea since 2004 [2]. None of the included studies provided zinc as part of the recommended treatment. To best estimate the effect probiotics could have on current home management of diarrhea, additional RCTs should be conducted to compare the currently recommended treatment, ORS, continued feeding, and zinc supplementation, with and without the addition of probiotics.

**Figure 3:**
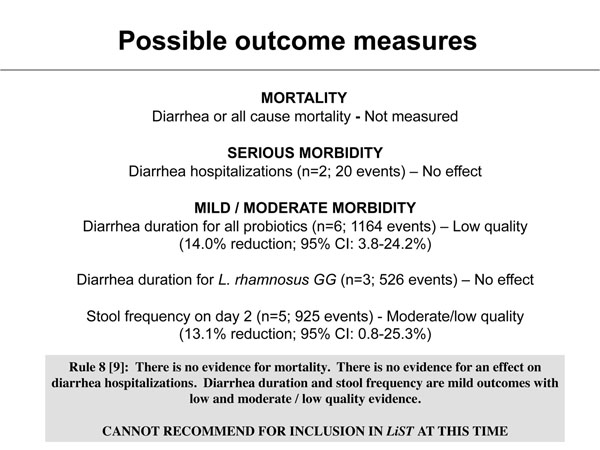
Application of standardized rules for choice of final outcome to estimate effect of probiotics on the reduction of diarrhea mortality

Interventions included in the LiST software are those that have been shown to reduce cause-specific mortality, or provide strong evidence of a reduction of severe morbidity among children less than five years of age [9]. We used the adapted GRADE technique [9] to assess the quality of evidence associated with the included studies by outcome. Despite being RCTs the combined quality score of included studies was low/moderate, primarily due to small sample size, inadequate blinding, and reduced generalizability (Tables 2 &3). Based on the lack of mortality data, no effect on severe morbidity (i.e., hospitalizations), and the low-moderate quality evidence for mild outcomes (i.e., diarrhea duration and stool frequency), there is insufficient evidence to conclude probiotics for the treatment of diarrhea will reduce diarrhea mortality, and thus at this time this intervention should not be included in *LiST* (Figure 3).

## Conclusions

This review highlights important implications for future research of the therapeutic effectiveness of probiotics, when compared with rehydration alone, for childhood diarrhea in LMIC. Community-based RCTs should be conducted in low- and middle-income countries to determine the effect of probiotic treatment, when compared with ORS, continued feeding, and zinc - the recommended treatment for community-acquired acute diarrhea among children <5 years of age. Furthermore, cost-effective analyses and qualitative studies should examine parental acceptance and access to probiotics to determine the feasibility of probiotic treatment in developing countries.

## List of abbreviations

CHERG: Child Health Epidemiology Reference Group; *LGG*: *Lactobacillus rhamnosus GG*; *LiST*: Lives Saved Tool; LMIC: Low- and middle-income countries; ORS: Oral rehydration salts; RCT: Randomized control trial.

## Competing interests

The authors declare that they have no competing interests.

## Authors' contributions

JAA led the review, abstraction, analysis and initial draft of manuscript; CLFW conceptualized the study and contributed to the analysis, interpretation, and manuscript preparation. RA conducted the initial literature search, contributed to the data abstraction, and reviewed the final manuscript. REB advised on the overall study design and methodology and contributed to the manuscript.

## Supplementary Material

Additional file 1 **Search terms for probiotics for the treatment of diarrhea literature search** Medical Subject Heading Terms (MeSH) and all fields search terms for *probiotics* and *diarrhea.* Click here for file

Additional file 2 **Standardized abstraction table of probiotics for the treatment of diarrhea data** Abstracted data by outcome and probiotic microorganism. Click here for file

Additional file 3 **Study characteristics of all included studies** Study characteristics including: treatment agent, treatment duration, standard of care, study location, age range of study population, and relevant outcomes. Click here for file

## Declarations

The publication costs for this supplement were funded by a grant from the Bill & Melinda Gates Foundation to the US Fund for UNICEF (grant 43386 to "Promote evidence-based decision making in designing maternal, neonatal, and child health interventions in low- and middle-income countries”). The Supplement Editor is the principal investigator and lead in the development of the Lives Saved Tool (LiST), supported by grant 43386. He declares that he has no competing interests.

This article has been published as part of *BMC Public Health* Volume 13 Supplement 3, 2013: The Lives Saved Tool in 2013: new capabilities and applications. The full contents of the supplement are available online at http://www.biomedcentral.com/bmcpublichealth/supplements/13/S3.
