# Supplementary material for: Systematic review of probiotics for the treatment of community-acquired acute diarrhea in children
Source: BMC Public Health. 2013 Sep 17;13(Suppl 3):S16. doi: 10.1186/1471-2458-13-S3-S16 (PMC3847198; doi:10.1186/1471-2458-13-S3-S16)
Supplement: Additional file 1 — Search terms for probiotics for the treatment of diarrhea literature search Medical Subject Heading Terms (MeSH) and all fields search terms for probiotics and diarrhea. [file 1471-2458-13-S3-S16-S1.pdf]

**DATABASE**	**PubMed**
**DATE OF ORIGINAL SEARCH**	**October 2011**
**TOTAL # RESULTS**	**25145**
**DATE RANGE FOR SEARCH**	**ALL AVAILABLE YEARS**
**LOCATION**	**ALL AVAILABLE LOCATIONS**
**HEALTH OUTCOME**	**Probiotics**

Search terms:
"Probiotics"[Mesh] OR "Probiotics"[All Fields] OR "Probiotic"[All Fields] OR "lactobacillus"[MeSH Terms] OR "lactobacillus"[Text Word] OR "lactobacilli"[Text Word] OR "betabacterium"[All Fields] OR "lactobacileae"[All Fields] OR "lactobacilleae"[All Fields] OR "lactobacteria" OR "Lactobacillus acidophilus"[Mesh] OR "Lactobacillus acidophilus"[All Fields] OR "Lactobacillus casei"[Mesh] OR "Lactobacillus casei"[All Fields] OR "Lactobacillus delbrueckii"[Mesh] OR "Lactobacillus delbrueckii"[All Fields] OR "Lactobacillus fermentum"[Mesh] OR "Lactobacillus fermentum"[All Fields] OR "Lactobacillus helveticus"[Mesh] OR "Lactobacillus helveticus"[All Fields] OR "Lactobacillus leichmannii"[Mesh] OR "Lactobacillus leichmannii"[All Fields] OR "Lactobacillus plantarum"[Mesh] OR "Lactobacillus plantarum"[All Fields] OR "Lactobacillus reuteri"[Mesh] OR "Lactobacillus reuteri"[All Fields] OR "Lactobacillus rhamnosus"[Mesh] OR "Lactobacillus rhamnosus"[All Fields]

**DATABASE**	**PubMed**
**DATE OF ORIGINAL SEARCH**	**October 2011**
**TOTAL # RESULTS**	**216408**
**DATE RANGE FOR SEARCH**	**ALL AVAILABLE YEARS**
**LOCATION**	**ALL AVAILABLE LOCATIONS**
**HEALTH OUTCOME**	**Diarrhea**

Search terms:
("Diarrhea"[Mesh] OR "Diarrhea" [All Fields] OR "Dysentery"[Mesh] OR "Dysentery"[All fields] OR "Gastroenteritis"[Mesh] OR "gastroenteritis"[All fields] OR "gastroenteritides"[All fields] OR "diarrhoeal disease"[All fields] OR "diarrhoeal diseases"[All fields] OR "enteritis"[All Fields] OR "Diarrheal Disease"[All fields] OR "Diarrheal Diseases"[All fields] OR "diarrheas"[All fields] OR "diarrhoea"[All Fields] OR "diarrhoeas"[All Fields] OR "gastrointestinal infection"[All Fields] OR "gastrointestinal infections"[All Fields] OR "Diarrhea, Infantile"[Mesh])

**DATABASE**	**PubMed**
**DATE OF ORIGINAL SEARCH**	**October 2011**
**TOTAL # RESULTS**	**2038**
**DATE RANGE FOR SEARCH**	**ALL AVAILABLE YEARS**
**LOCATION**	**ALL AVAILABLE LOCATIONS**
**HEALTH OUTCOME**	**Diarrhea + Probiotics**

Search terms:
("Diarrhea"[Mesh] OR "Diarrhea" [All Fields] OR "Dysentery"[Mesh] OR "Dysentery"[All fields] OR "Gastroenteritis"[Mesh] OR "gastroenteritis"[All fields] OR "gastroenteritides"[All fields] OR "diarrhoeal disease"[All fields] OR "diarrhoeal diseases"[All fields] OR "enteritis"[All Fields] OR "Diarrheal Disease"[All fields] OR "Diarrheal Diseases"[All fields] OR "diarrheas"[All fields] OR "diarrhoea"[All Fields] OR "diarrhoeas"[All Fields] OR "gastrointestinal infection"[All Fields] OR "gastrointestinal infections"[All Fields] OR "Diarrhea, Infantile"[Mesh]) AND ("Probiotics"[Mesh] OR "Probiotics"[All Fields] OR "Probiotic"[All Fields] OR "lactobacillus"[MeSH Terms] OR "lactobacillus"[Text Word] OR "lactobacilli"[Text Word] OR "betabacterium"[All Fields] OR "lactobacileae"[All Fields] OR "lactobacilleae"[All Fields] OR "lactobacteria" OR "Lactobacillus acidophilus"[Mesh] OR " acidophilus"[All Fields] OR "Lactobacillus casei"[Mesh] OR " casei"[All Fields] OR "Lactobacillus delbrueckii"[Mesh] OR " delbrueckii"[All Fields] OR "Lactobacillus fermentum"[Mesh] OR " fermentum"[All Fields] OR "Lactobacillus helveticus"[Mesh] OR " helveticus"[All Fields] OR "Lactobacillus leichmannii"[Mesh] OR " leichmannii"[All Fields] OR "Lactobacillus plantarum"[Mesh] OR " plantarum"[All Fields] OR "Lactobacillus reuteri"[Mesh] OR " reuteri"[All Fields] OR "Lactobacillus rhamnosus"[Mesh] OR " rhamnosus"[All Fields]) NOT (animals[mh] NOT humans[mh])

*The above search terms were used in the original search (October 2011) and the updated search (December 2012).

Supplementary table. ***Excluded full-text articles by reason for exclusion***

Reason for exclusion	Number of studies	Percentage (%)
Antibiotics	9	7.1
Breastfeeding	15	11.9
Did not evaluate probiotics alone	8	6.3
Excluded specific etiologies	13	10.3
No proper control group	13	10.3
Not RCT	22	17.5
Not acute diarrhea or diarrhea not defined	22	17.5
Study population >5 yrs	8	6.3
Poor methodological quality	16	12.7
**Total excluded based on full-text**	**126**	
