# Supplementary material for: Systematic review of probiotics for the treatment of community-acquired acute diarrhea in children
Source: BMC Public Health. 2013 Sep 17;13(Suppl 3):S16. doi: 10.1186/1471-2458-13-S3-S16 (PMC3847198; doi:10.1186/1471-2458-13-S3-S16)
Supplement: Additional file 3 — Study characteristics of all included studies Study characteristics including: treatment agent, treatment duration, standard of care, study location, age range of study population, and relevant outcomes. [file 1471-2458-13-S3-S16-S3.pdf]

Author	Study arms					Standard of care	Location	Age	Outcomes
	Intervention			Control					
	n	*Probiotic micro-organism* (Dosage)	Duration & method of treatment	n	Placebo				
Boudraa [12]	56	*L. bulgaricus & S. thermophilus* ($2x10^8$ CFU/g)	180 mL/kg/day infant formula given after initial oral rehydration	56	Infant formula acidified with lactic acid to match yogurt pH	ORS & continued feeding	Algeria	3-24 m	Diarrhea duration
Canani [13]	100	*Lactobacillus rhamnosus GG* ($6x10^9$ CFU/dose)	2/day for 5 days in 20 ml water	92	Not described	ORS, then formula containing lactose or cow's milk	Italy	3-36 m	Diarrhea duration<br>Stool frequency<br>Hospitalizations
	91	*S. boulardii* ($5x10^9$ live micro-organisms/dose)	2/day for 5 days in 20 ml water	92					
	100	*Bacillus clausii* (10^9 CFU/dose)	2/day for 5 days in 20 ml water	92					
	97	*L. bulgaricus, L. acidophilus, Streptococcus thermophilus, B. bifidum* ($5x10^8$- 10^9 CFU /dose)	2/day for 5 days in 20 ml water	92					
	91	*Enterococcus faecium* ($7.5x10^7$ CFU/dose)	2/day for 5 days in 20 ml water	92					

Cetina-Sauri [18]	65	*S. boulardii* (200 mg/dose)	Every 8 hours in 5ml of cold liquid	65	200mg glucose in 5ml cold liquid	WHO oral electrolytes & continued feeding	Mexico	3–36m	Stool frequency
Costa-Ribeiro [14]	61	*Lactobacillus GG* ($10x10^9$ CFU/day)	1/day w/oral electrolyte solution	63	Inulin	ORS	Brazil	1-24 m	Diarrhea duration
Lee [15]	50	*Lyophilized L. acidophilus and B. infantis* ($3x10^9$ CFU of each organism/day)	1/day for 4 days	50	Not described	Parenteral rehydration	Taiwan	6-60 m	Diarrhea duration<br>Stool frequency
Misra [16]	105	*Lactobacillus GG* ($1x10^9$ CFU/dose)	1/day for 10 days	105	Identical placebo (crystalline microcellulose)	Not described	India	< 36 m	Diarrhea duration
Rafeey [19]	40	*L.acidophilus* ($5x10^9$ CFU /capsule)	2 capsules/ day	40	Not described	WHO fluid therapy, no other fluids or food	Iran	$\leq$ 60 m	Stool frequency
Veereman-Wauters [17]	Diarrhea duration: 25<br>Stool frequency: 22	*Lactobacillus GG, L. acidophilus, L. casei, L. plantarum, B. infantis* ($3x10^9$ CFU /capsule)	3 capsules/ day for 9 days	22	Identical placebo (maltodextrine)	ORS & continued feeding	Belgium	1-60 m	Diarrhea duration<br>Stool frequency<br>Hospitalizations
